# The Role of Government Innovation Support in the Process of Urban Green Sustainable Development: A Spatial Difference-in-Difference Analysis Based on China’s Innovative City Pilot Policy

**DOI:** 10.3390/ijerph19137860

**Published:** 2022-06-27

**Authors:** Hongge Zhu, Zhenhuan Chen, Shaopeng Zhang, Wencheng Zhao

**Affiliations:** 1 College of Economics and Management, Northeast Forestry University, Harbin 150040, China; honggebill@nefu.edu.cn (H.Z.); czh2017@nefu.edu.cn (Z.C.); 2 School of Management, Harbin Institute of Technology, Harbin 150040, China; 3 Institute of Finance and Economics, School of Urban and Regional Science, Shanghai University of Finance and Economics, Shanghai 200433, China; zhaowencheng@163.sufe.edu.cn

**Keywords:** government innovation support, innovative city pilot policy, green sustainable development, green technology innovation, spatial difference-in-difference model, China

## Abstract

The role of government support in sustainable urban development has always been a research topic of scholars, but research focusing on the relationship between government innovation support and urban green sustainable development is still relatively rare. This article uses China’s innovative city pilot policy (ICPP) to represent the innovation support provided by the government and address the interaction mechanism and the spatial spillover effect of China’s innovative city pilot policy (ICPP), green technology innovation (GTI), and green sustainable development performance (GSDP) with the support of the mediating effect model and the spatial econometric model. Based on panel data of 24 cities in the Yangtze River Delta urban agglomeration from 2001 to 2020, this paper establishes an evaluation index system of green sustainable development performance (GSDP), measuring with the SBM directional distance function based on the undesired output. This paper adopts the spatial difference-in-difference model (SDID) to study the impact mechanism of the ICPP on the GSDP in the Yangtze River Delta. The results show that (i) there is a positive spatial spillover effect of GSDP in the urban agglomeration of the Yangtze River Delta urban agglomeration; (ii) ICPP has a significantly positive effect on GSDP, as verified by several robustness checks; (iii) green technology innovation plays a partial mediating effect in the relationship of the ICPP and GSDP.

## 1. Introduction

Improving human well-being, protecting the environment, and promoting sustainable development through green development are key 2030 Global Sustainable Development goals [1]. However, China’s economic growth has brought with it serious problems such as high energy consumption, high emissions, and high pollution, and the ecological environment has been severely damaged [2,3]. In this case, in order to meet the people’s yearning for a better life, adhering to green development has become an inevitable choice for China’s sustainable development. The realization of green development is an important driving force for promoting the process of China’s ecological civilization construction and maintaining the sustainable development of China’s economy and society [4]. At the same time, green sustainable development is also an important part of building a modern economic development system. In the process of China’s economic transformation from high-speed growth to high-quality development, green sustainable development is the fundamental policy to solve the problem of environmental pollution, and a key step in the promotion of the coordinated evolution of high-quality development and ecological environment protection [5,6]. Nowadays, green sustainable development is not only an important issue concerning government and society but also a hot topic focused on by many scholars.

The concept of green sustainable development has been put forward based on critiques of the traditional development model. For example, Daly and Cobb [7] think that green sustainable development is an economic development mode that does not lead to unsustainable economic development due to the depletion of natural resources, and the green development model is conducive to solving the problems of low energy utilization efficiency and ecological environment pollution in economic development. On this basis, Giddings et al. [8] proposed that green sustainable development is generally represented by the intersection of environment, society, and economy. Therefore, green sustainable development should ensure that while the economy is growing rapidly, the ecological environment is also significantly improved, and people can enjoy high-quality living conditions. Scholars have attained rich research achievements on the current situation and problems of China’s green sustainable development. For example, Zeng and Bi. [9] measured and analyzed the condition of China’s green sustainable development at the provincial level, and they argue that China’s green sustainable development requires steady progress before it can feed back into economic growth. Wang et al. [10] evaluated the green sustainable development level of nine cities in the Pearl River Delta and found that the green sustainable development work in these nine cities had made some progress, but there were still some shortcomings in energy savings and emissions reduction. In addition, He et al. [11] believe that China’s industrial development has not yet fully met the requirements of green sustainable development, and how to measure the performance of green sustainable development should be the focus of current research. Conceptually, green sustainable development performance (GSDP) is the measurement of the completion and implementation efficiency of urban green sustainable development. Measurement methods include the AHP method, principal component analysis, entropy weight method, and data envelope analysis (DEA) [12]. In recent years, the use of DEA to measure GSDP has become a mainstream trend in academia. In particular, the SBM directional distance function incorporates undesired outputs such as environmental pollution, avoiding the defect that traditional DEA only considers economic output [13].

The city is the basic space carrier for human beings to engage in economic activities, and it is also because of the influence of people’s production and lives that urban industrial pollution, domestic waste, and locomotive exhaust gas and other environmental problems are becoming increasingly serious. Therefore, the city has become an important focus for strengthening pollution prevention and promoting green development. Many scholars have found that technological innovation has a positive effect on sustainable urban development [14,15], especially the increasingly prominent role of green technology innovation in environmental pollution control [16]. Green technology innovation (GTI) is an innovation that can both bring benefits to enterprises and reduce adverse impacts on the environment. It includes technological innovations in energy conservation, pollution prevention, waste recycling, green product design, and environmental management [17]. Therefore, in order to promote the process of urban green sustainable development, central and local governments have issued relevant policies to support the development of urban innovation activities and the construction of innovation systems [5,13,18]. In 2008, Shenzhen took the lead in implementing the innovative city pilot policy (ICPP). Since then, the Chinese government has promulgated the “Guiding Opinions on Promoting the Pilot Work of Innovative Cities” and the “Guidelines for Building an Innovative City” beginning in 2010 and 2016, respectively, which further clarifies the goals and implementation plans of the ICPP. As of 2022, a total of 103 cities have entered the ICPP pilot list, and the ICPP has now become a representative government innovation support policy in China. The ICPP refers to the policy exploration of carrying out urban innovation activities and improving urban innovation ability under the support of the government so that pilot cities can develop into innovative cities with strong independent innovation abilities with scientific and technological support and which play a leading role [19]. In the process of implementing the pilot policy for innovative cities, studies have tried to bring the concept of eco-city into the category of innovative city construction and to explore the urban green sustainable development mode of achieving economic sustainability and natural ecological health by giving play to the positive role of urban innovation in environmental protection [20,21,22]. Thus, under a policy background of the innovative city pilot project, will government innovation support positively affect urban green sustainable development? If so, what is the transmission mechanism of this effect? At present, the existing research does not provide a definite answer.

The Yangtze River Delta urban agglomeration is one of the most developed regions in China. However, due to the influence of the extensive development mode and the long-term natural ecology of the Yangtze River, the environmental pollution phenomenon is relatively serious, and the overall level of ecological efficiency is low [20,23]. At the same time, the Yangtze River Delta region is also the backbone of China’s technological innovation. With the support of national policies, the Yangtze River Delta urban agglomeration is the area with the highest intensity of implementation of the ICPP. Nineteen cities have been approved for innovative city pilot projects, and they are the pioneers of China’s innovative city construction [24]; see Appendix A for the full list. Therefore, this paper took the Yangtze River Delta urban agglomeration as the research object and used urban panel data from 2001 and 2020 to evaluate the green sustainable development performance (GSDP). On this basis, we aimed to clarify the effect of the ICPP, which represents government innovation support for GSDP, and its influence mechanism to provide a decision-making reference for the construction of China’s innovative country and the promotion of GSDP.

The remaining structure of this paper is as follows. In Section 2, the research gaps and possible contributions of this study are presented based on a review of the existing literature. Section 3 discusses the materials and methods used, mainly the spatial difference-in-difference model (SDID) settings, selection of variables, and data sources. Section 4 presents the empirical results including the results of the spatial autocorrelation test, parallel trend test, benchmark regression, and robustness checks. Section 5 is the mechanism analysis, which mainly included the analysis of the mediating mechanism, the settings of the mediation effect model, and the test results. Section 6 provides the discussion, and Section 7 concludes the paper.

## 2. Literature Review

Green sustainable development is a concept that carefully considers the economic, social, and environmental impacts of development; thus, it is vital to establish a reasonable evaluation index system to measure and analyze green development performance. From the literature research, most scholars made improvements based on existing achievements. Among them, the economic, social, environmental, and institutional four-system framework model constructed by the United Nations Commission on Sustainable Development is a more comprehensive evaluation index system for GSDP at this stage. In addition, many scholars are also actively carrying out research on green economic accounting and green development capacity measurement such as Vogtlander et al. [25], Moussiopoulos et al. [26], and Kim et al. [27]. Other scholars have focused on methods to realize green sustainable development. For example, Li et al. [28] not only established a theoretical framework but also formulated an implementation mechanism for the current status and problems of China’s green sustainable development. Other scholars take the influencing factors of green sustainable development as their research topic such as environmental regulation [21], green organizational identity [29], the constraints of haze [30], financial agglomeration [31], and the high-tech industrial scale [23]. According to the research objects, most scholars conduct empirical research at the national level [28,32], provincial level [9,30], and industrial level [33,34]. Through a literature review, it was found that empirical research based at the urban level is relatively rare, and the research on the condition and influencing factors of GSDP need to be further supplemented.

How to rely on innovation to drive green sustainable development is a hot issue in academic circles. As is known, technological innovation not only requires sufficient R&D personnel and funding but also requires a good innovation milieu to ensure the orderly development of scientific and technological innovation activities [35]. Innovation milieu is a multidimensional concept that not only includes the complex network relationships among the innovation subjects but also the systematic institutional arrangement [36]. The innovation system theory believes that regional market, culture, system, and other innovation environment factors play an important role in the agglomeration of innovation elements. Especially, the government’s investment in innovation activities and policy support (such as macroeconomic policy, science and technology policy, and industrial policy) are an important source of power to promote technological innovation [37], and the innovation absorption and application capabilities of different regions will also directly affect the agglomeration level of local innovation elements [38]. In addition, research on the geography of innovation has found that spatial or geographic proximity has a significant impact on technological innovation [36,39,40,41]. Therefore, the government should try to create a good innovation environment for local cities and drive the innovation capacity of surrounding cities through the spatial spillover effect, which is crucial for realizing regional sustainable development.

In recent years, the role of the government in the construction of regional innovation systems has gradually been paid increasing attention by scholars. Most of them found that government innovation support is conducive to promoting regional innovation capabilities [42,43,44]. In China, the construction of an innovative country is not only the government’s strategic goal but also includes the fundamental policy support provided by the government. In the process of innovative country construction, the implementation of the ICPP is through typical government innovation support. Innovative city refers to a city whose development is mainly driven by innovative elements, such as technology, knowledge, manpower, culture, and system, and plays a leading role for other cities in the region. In the related research on innovative cities, we found that there were two main expressions regarding innovative city [45,46]: one is “the creative city” and the other is “the innovation city”. There is a difference between creativity and innovation: one emphasizes the development of ideas and the other puts these ideas into practice. Therefore, the former emphasizes solutions to complex urban problems by proposing creative methods, while the latter emphasizes the application of innovative methods to solve complex problems in urban development [47]. In the research on the construction of innovative cities in China, scholars mostly define the concept of innovative cities based on the driving force of “innovation” elements for urban development [48]. The elements of an innovative city mainly include innovation resources, innovation subject, innovation milieu, and innovation achievement [47]. Among them, innovation achievement is the direct embodiment of urban innovation ability [33]. Some scholars have also pointed out that the achievement of urban innovation is mainly carried out by innovation subjects, such as enterprises, universities, and research institutes, in a positive innovation milieu so as to transform the urban innovation resources into output that is conducive to the release of urban innovation [49,50]. 

Thus far, the existing literature lacks a systematic theoretical interpretation of the impact of government innovation support on green and sustainable development. The theoretical basis closely related to this topic is the impact of innovation on the ecological environment, and most scholars support the view that innovation can promote the ecological environment. For example, Roy [51] argued that in national economic development, scientific and technological innovation is the only way to promote the country from an industrial society to an ecological society. Bryan [52] emphasized the adaptability of the innovation milieu among the public. He believed that a positive innovation milieu was a strong aspect in helping the ecological environment achieve sustainability. The research by Charmondusit et al. [53] showed that a positive innovation atmosphere has a promoting impact on ecological efficiency, which is conducive to improving sustainable development and cleaner production. In addition, some studies found that innovation is conducive to reducing haze pollution and improving environmental quality [54,55]. On this basis, many scholars have gradually extended their focus to the impact of technological innovation on ecological economy or green sustainable development. Ehresman [56] integrated relevant concepts related to green sustainable development in the existing research based on the perspective of environmental justice and found that there was a positive correlation between national innovation capacity and green development performance. Feng et al. [34] showed that technological innovation had a positive impact on the quality of urban development and was conducive to improving the green and sustainable development of cities. 

The above literature is important for understanding how innovation drives green sustainable development, but these studies were mainly carried out from the perspective of the innovation level rather than government innovation support. In fact, like the level of innovation, government innovation support based on innovation policy is also an important part of the construction of the national innovation system [57]. The establishment of the ICPP in China provides a valuable opportunity for assessing the impact of government innovation support on green sustainable development. With this policy background, the construction process of an innovative city gradually transits from the exploratory development stage to the diffuse development stage [15,48]. The period from 2005 to 2010 was the exploratory development stage of the ICPP. During this period, cities such as Beijing and Shenzhen took the lead in putting forward the slogan of building an innovative city. Subsequently, the Chinese government officially announced that 41 cities, including Shenzhen, Beijing, Tianjin, Shanghai, and Nanjing, had become ICPP pilot cities. From 2011 to now, the ICPP has had diffuse development stages, and the ICPP has spread to 103 cities. Meanwhile, the Chinese government has carried out a systematic evaluation of the ICPP’s construction effect [58]. Most scholars believe that innovative city construction and urban green sustainable development often complement each other, and both are driven by technological innovation [21,48,59]. Some scholars have proposed that green sustainable development has a positive impact on urban innovation capacity and competitive advantage [60,61]. However, more scholars have focused on the impact of urban innovation on green sustainable development. For instance, Lorek [62] believed that innovative cities tend to change toward the direction of a green economy, which cannot only effectively reduce energy consumption but also push forward the process of green sustainable development. The findings of Jouvet and Perthuis [63] showed that the rapid development of urbanization is an important reason for environmental pollution, which is contrary to the strategic goal of green sustainable development. However, urban innovative construction can more reasonably promote the development of urbanization and effectively alleviate the negative effect on green sustainable development. Furthermore, some scholars have found through empirical research that urban innovation systems and capacity development have a positive influence on GSDP [13,64]. To sum up, the positive role of government support policies and technological innovation in promoting sustainable development has been confirmed by an increasing number of studies [65,66,67]. 

Through the literature review above, we found that scholars have achieved fruitful research results in this field, but there are still some research deficiencies. Firstly, previous studies on the connotation, evaluation index system, and measurement method of green sustainable development has matured, but many studies mainly focus on the innovation level at the national, provincial, or industrial level and lack evaluation of the impact of innovation policies on green sustainable development at the city level. Secondly, in research on the treatment effect of the ICPP, scholars mostly used the DID model to conduct empirical research. However, the traditional DID model often ignores the spatial correlation and spatial heterogeneity between the research areas, which easily leads to deviations in the analysis results. Thirdly, although many scholars have studied the relationship between urban innovation and GSDP, research focusing on the treatment effect of the ICPP on GSDP is still relatively rare, and studies clarifying the impact mechanism between the two are even rarer.

**Figure 1:**
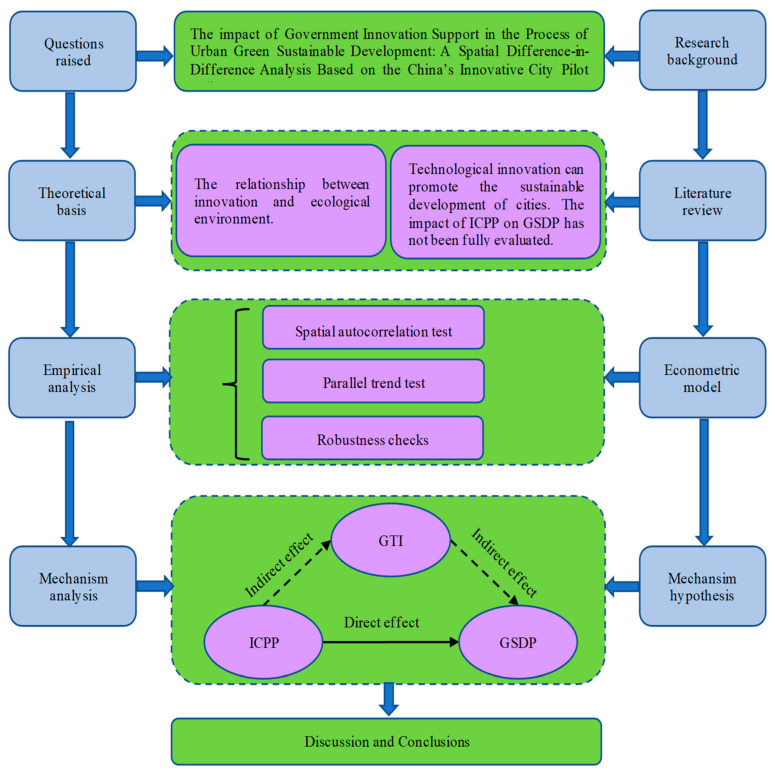
The framework and technical route of this study.

In order to fill the research gaps, the possible contributions of this paper are as follows. Firstly, considering that the Chinese government’s high-quality development strategy emphasizes a win–win situation between economic development and ecological environment, this article aimed to design a green sustainable development evaluation system suitable for China’s national conditions and to measure GSDP based on China’s development situation. Secondly, this study took the Yangtze River Delta urban agglomeration as the research object and used urban panel data to empirically analyze the effect of the ICPP on GSDP. Third, this study adopted the SDID model to bring the spatial effect into the impact mechanism so that we could decrease the estimation error and obtain more accurate regression results. Finally, this study verified the mediating effect of green technology innovation in the relationship between the ICPP and GSDP through theoretical and empirical analysis. As shown in Figure 1, the research framework of this paper is displayed more intuitively and in detail.

## 3. Materials and Methods

### 3.1. Model

Considering that the difference-in-difference (DID) model has its unique advantages in the evaluation and analysis of policy effects [68], this paper selected the DID model to study the treatment effects of the ICPP on GSDP. In addition, from the perspective of spatial econometrics, urban innovation may have spillover effects on adjacent spatial geographical units [33,69]. Particularly, the ICPP not only has an impact on the GSDP of the city but may also have an impact on the GSDP of surrounding cities. Moreover, if the traditional DID model is used alone, the spatial spillover effect on GSDP may be ignored. In order to effectively reflect the spatial effect of the ICPP on GSDP, this paper used the SDID model for empirical analysis. The SDID model was set as follows:

(1) $${\mathrm{GSDP}}_{\mathrm{it}}=\sum_{\mathrm{it}}^{\mathrm{NT}}\rho W\times {\mathrm{GSDP}}_{\mathrm{it}}+\beta_{0}+\sum_{k=1}^{K}X_{\mathrm{it},k}\beta_{k}+{\mathrm{ICPP}}_{\mathrm{it}}\beta_{k+1}+\mu_{\mathrm{it}}+\nu_{\mathrm{it}}+\varepsilon_{\mathrm{it}}$$

In Equation (1), ${\mathrm{GSDP}}_{\mathrm{it}}$ is the dependent variable used in this paper, representing the performance of green sustainable development; i = 1, 2, …, N (N = 24), representing 24 prefecture-level cities in the Yangtze River Delta urban agglomeration; t = 1, 2, …, T (T = 20), which represents the time stage when the ICPP was implemented; $X_{\mathrm{it},k}$ represents the k control variables selected in this paper, k = 1, 2, …, K (K = 6).

${\mathrm{ICPP}}_{\mathrm{it}}$ is the treatment effect parameter, a dummy variable for the implementation of the innovative city pilot policy. That is, if the time variable is the year when the policy was implemented in the city or beyond, ${\mathrm{ICPP}}_{\mathrm{it}}\;\;$ = 1; otherwise, ${\mathrm{ICPP}}_{\mathrm{it}\;}$ = 0. W represents the spatial weight matrix, the setting principle of which is as follows: if two cities are adjacent in spatial location, the matrix element is 1; otherwise, it is 0, namely, the spatial adjacency matrix [21]. ρ represents the spatial correlation coefficient of the spatial econometric model; $\beta_{k}$ reflects the regression coefficient of the model; $\mu_{\mathrm{it}}$ represents the spatial-fixed effects; $v_{\mathrm{it}}$ represents the time-fixed effects; $\varepsilon_{\mathrm{it}}$ denotes the random error term.

### 3.2. Variables

#### 3.2.1. Dependent Variable

Based on a framework model of economy, resources, energy, and environment, combined with the research findings of Moussiopoulos et al. [26], Yang et al. [23], Li et al. [59], Yuan et al. [31], and Cheng and Ge [32], this paper established an evaluation index system suitable for China’s national conditions. The evaluation index system of GSDP contained five input indicators, which were labor, capital, technology, water resources, and electric energy, and two output indicators, which were the desired and undesired outputs. Specifically, labor input was characterized by the number of employees in urban units; capital input was depreciated by the gross domestic product (GDP) deflator to the fixed asset investment based on the perpetual inventory method, and the depreciation rate was 9.6%. Technology input was measured by science and technology expenditure; water resources input was reflected by the total water supply; electric energy input was represented by society’s entire electricity consumption. In addition, the desired output was characterized by the regional GDP and the green area of built-up areas; the undesired output was measured by industrial wastewater emissions, sulfur dioxide emissions, and dust emissions.

The detailed index system is shown in Table 1. In this paper, the SBM directional distance function based on the undesired outputs was used to measure the green total factor productivity, which reflects the GSDP of the Yangtze River Delta urban agglomeration.

#### 3.2.2. Independent Variable

The core independent variable of this paper was the ICPP, that is, the treatment effect parameter in SDID model. When selecting other independent variables, this paper referred to research on the influencing factors of green economy or green sustainable development by Feng and Chen [21], Yuan et al. [31], Liu et al. [24], and Yuan et al. [1], and controls other variables that may affect GSDP, mainly foreign direct investment (FDI), human capital level (HCL), labor structure (LAS), information infrastructure (INI), R&D intensity (RDI), and urbanization level (URL). Among them, we used the proportion of urban foreign direct investment on the mainland, Hong Kong, Macao, and Taiwan and the gross domestic product to measure FDI. We used the proportion of the number of college students to the total urban population to measure HCL, and the ratio of the number of highly skilled laborers to the number of low-skilled laborers was used to measure LAS. The proportion of the total amount of post and telecommunications business and gross domestic product was used to measure INI. Moreover, we used the ratio of R&D investment to gross domestic product to measure RDI and used the proportion of urban population and total urban population at the end of the year to measure URL. Table 2 shows the descriptive analysis results for each variable, the correlation analysis results with GSDP, and unit root test results.

Observing the correlation analysis results, we can see that the correlation coefficient between the ICPP and GSDP was positive at the significance level of 1%. This result can preliminarily judge that the ICPP can effectively improve GSDP, but it still needs to be further tested by regression analysis. The unit root test (IPS test and LLC test) results show that the data used in this study are relatively stable and could be used as panel data for regression analysis.

### 3.3. Data Sources

The Yangtze River Delta urban agglomeration was the research object of this paper. Among its 26 prefecture level cities, 19 cities have been approved as an innovative city pilot, with high pilot intensity and active urban innovation activities; see Appendix A for a full list of treatment groups. However, due to the missing data of Ningbo in Zhejiang Province and Taizhou in Jiangsu Province, the two cities were excluded from the sample. The list of cities in the control group included Taizhou, Zhoushan, Tongling, Anqing, Chuzhou, Chizhou, and Xuancheng. The sample period was 2001–2020; thus, the total sample size was 24 × 20 = 480. The data used in this paper were mainly from the China Urban Statistical Yearbook, China Regional Economic Statistical Yearbook, China Urban Construction Statistical Yearbook, and regional city statistical yearbooks. It should be noted that in order to eliminate the influence of price factors, this paper used the price index of 2001 as the base period to adjust the variables related to value to form the corresponding constant value. The linear interpolation method was used to supplement the missing or singular values of some indicators.

## 4. Empirical Results

### 4.1. Spatial Autocorrelation Test

Anselin (1992) believes that spatial effects (i.e., spatial autocorrelation and spatial heterogeneity) are often overlooked by scholars and that considering spatial effects in empirical research may bring new insights [70]. Before spatial econometric analysis, a spatial autocorrelation test is generally used to verify whether an element has a spatial spillover effect within adjacent spatial units [71]. According to Anselin (1995), the Global Moran’s I index can measure whether there is interdependence between variables in space. The value of the Global Moran’s I was between −1 and 1, and it is generally believed that the larger the absolute value, the stronger the spatial correlation. The advantage of using Global Moran’s *I* is that the results are less likely to deviate from the normal distribution [72,73]. To conduct the spatial autocorrelation test, we adopted Global Moran’s I to measure the spatial characteristics. We measured the Global Moran’s I of GSDP using the Stata, version 16, software. In Table 3, we can see that there was positive spatial correlation with GSDP from 2001 to 2020. The results show that GSDP had a significantly positive spatial spillover effect at the 1% level in most years.

### 4.2. Parallel Trend Test

The primary premise of using the SDID model is that the treatment group and the control group of the research samples must have a parallel trend, and this parallel trend cannot change significantly with time [74]. In order to test whether GSDP passes the parallel trend hypothesis, this paper judged the evolution trend of the average GSDP of the Yangtze River Delta urban agglomeration before 2009. The reason why we chose the average value of GSDP before 2009 for the parallel trend test was that the first cities in the Yangtze River Delta urban agglomeration, such as Nanjing, Hangzhou, and Hefei, became the innovative city pilots in 2009.

**Figure 2:**
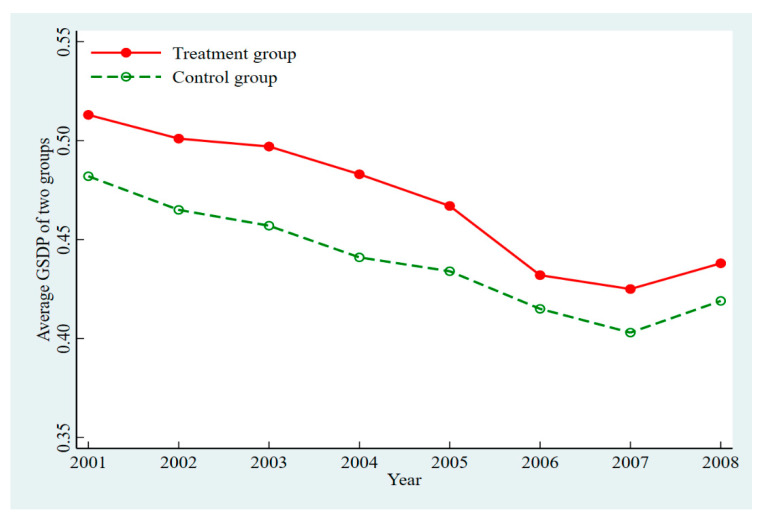
The average GSDP of the two groups in the Yangtze River Delta urban agglomeration from 2001 to 2008.

As shown in Figure 2, before 2009, although the average GSDP of the treatment group and the control group had some differences, the degree of the difference between the sample groups was relatively stable. In other words, the evolutionary trend of the two groups was basically the same. Therefore, it can be concluded that the GSDP of the treatment group and the control group passed the parallel trend hypothesis test before the implementation of the ICPP, which meets the premise of the SDID model. In addition, the test results also show that this study has high credibility. Moreover, we can see that the average GSDP for both of the groups were in an overall declining trend from 2001 to 2008, which indicates that in the early development stage of the Yangtze River Delta urban agglomeration, the extensive development mode was the main mode, and the emphasis of green sustainable development was at a low level.

### 4.3. Benchmark Results

In this paper, the SDID model was used to study the impact of the ICPP on GSDP. Only the dummy variable, TR, representing whether a city implements a policy, the dummy variable, TP, representing the policy implementation year, and the interaction of the two variables in the ICPP were added in Model 1, while the related control variables were added to Model 2 based on Model 1. The regression results of the models are shown in Table 4.

Firstly, from the regression results of Models 1–2 shown in Table 4, we can see that the treatment effect of the ICPP had a significantly positive impact on the GSDP in the Yangtze River Delta urban agglomeration at the 1% level. Secondly, from the results of the spatial spillover effects in Table 4, the spatial correlation coefficients (rho) of the two models were significantly positive at the 1% level, indicating that GSDP had a positive spatial spillover effect in similar cities in the Yangtze River Delta urban agglomeration. There are two classic explanations in the field of environmental pollution control. On the one hand, the Porter hypothesis argues that appropriate environmental regulation can encourage enterprises to carry out more innovative activities, and these innovations will improve the productivity of enterprises, thereby offsetting the cost of environmental protection and improving enterprise competitiveness [75]. On the other hand, the pollution paradise hypothesis states that enterprises in pollution-intensive industries tend to be established in countries or regions with relatively low levels of environmental regulation [76]. This means that the Yangtze River Delta region’s GSDPs may be more in line with the pollution haven hypothesis when adjacent cities take more strict environmental policies to control environmental pollution; it also encourages local cities to enact corresponding environmental regulation policy and promote pollution-intensive industry enterprises to select shifts in production activities to the periphery, thereby enhancing the overall green sustainable development level of the Yangtze River Delta urban agglomeration.

It was further found that after the implementation of the ICPP, not only the GSDP of the implemented cities was greatly improved, but also the green sustainable development undertakings of neighboring cities without implementation realized the spillover effect with the institutional dividend of the ICPP. Observing the results of the control variables in Model 2, FDI had a significantly negative influence on GSDP at the 5% level, which verifies the “pollution paradise” hypothesis and the negative impact of rapid urban expansion [15]. In addition, LAS, INI, and RDI had a significantly positive influence on GSDP at the 5% level, which indicates that the higher the proportion of highly skilled labor, the more perfect the information infrastructure construction, and the more R&D investment, the stronger the promotion of GSDP.

### 4.4. Robustness Checks

In the benchmark regression analysis, we conducted the SDID model based on the spatial adjacency matrix to study the effect of the ICPP on GSDP, which was measured with the SBM directional distance function based on the undesired outputs. In order to verify the robustness of the benchmark results, we performed several robustness checks. 

Firstly, the spatial distance matrix was used for the SDID model to substitute the spatial adjacency matrix. Secondly, we replaced the SBM directional distance function with the epsilon-based measure (EBM) model, for the reason that the EBM model, combined with the CCR model with radial factors and the non-radial SBM model with slack variables, can eliminate the bias of the measurement results and further improve the accuracy of the model. Thirdly, we adopted counterfactual tests to conduct regression analysis by advancing the time when the innovative city pilot was approved by 1 year. If the ICPP was not significant at this time, the robustness of the benchmark regression results was verified, otherwise, there were other systematic errors in the results. The results of robustness checks are shown in Table 5. 

As shown in Table 5, Model 3 was used for the first robustness check, Model 4 was used for the second check, and Model 5 was used for the third. It can be seen from both Model 3 and Model 4 that the ICPP had a significantly positive impact on GSDP at the 1% level. However, the ICPP was not significantly positive correlated with GSDP in Model 5. Therefore, we can conclude that the robustness of the benchmark results was verified.

## 5. Mechanism Analysis

### 5.1. Theoretical Analysis of the Mediating Effect

Based on the SDID regression results, the positive impact of the ICPP on the GSDPs in the Yangtze River Delta urban agglomeration was confirmed, but the “black box” of the treatment effect was not further illuminated. Therefore, the type of transmission mechanism that exists between the ICPP and GSDP became the key problem to be solved in this study. After referring to the related research, this paper believes that the impact mechanism of innovative city construction on green sustainable development mainly followed a path.

Particularly, some scholars believe that urban development under the guidance of an innovative policy environment pays special attention to green economic performance and is more committed to promoting economic growth and green sustainable development with green innovation [14,53,77,78]. In addition, Bekhet and Latif [79] found that green technology innovation can promote the quality of environmental governance institutions in Malaysia, and the interaction between them can further promote the green sustainable development of Malaysia. Song and Wang [80] hold a similar view; they argue that green technology innovation can improve resource utilization efficiency and reduce energy consumption in order to realize green development by optimizing resource allocation and releasing ecological value. The research conclusions of Feng and Chen [21] showed that green technology innovation can significantly promote industrial green development based on the spatial Durbin model and further improve the urban green development process. Referring to the above research achievements, this paper put green technology innovation into the research framework to explore whether green technology innovation plays a mediating role between the ICPP and GSDP. 

### 5.2. Mediating Effect Model

Based on the above analysis of the transmission mechanism between the ICPP and GSDP, the mediating variable selected in this paper was green technology innovation (GTI). As GTI is a branch of the field of technological innovation, it will lead to great errors and ambiguity when defining the number of patents. Therefore, this paper used the classification of green patents, based on “pollution control, energy conservation and emissions reduction, recycling, new energy and alternative energy sources, environment, material, green construction, green agriculture, green, green management of forestry” as keywords when searching the State Intellectual Property Office of the Platform to obtain the patent data for China’s Yangtze River Delta urban agglomerations in 2001–2020. In order to reduce the influence of heteroscedasticity, this paper measured urban GTI by the logarithm of the green patents. Therefore, in order to verify whether GTI had a mediating effect on the impact mechanism of the ICPP and GSDP, this paper used the mediating effect model for quantitative verification. The expressions of the model’s settings are as follows:

(2) $${\mathrm{GSDP}}_{\mathrm{it}}=\sum_{\mathrm{it}}^{\mathrm{NT}}\rho W\times {\mathrm{GSDP}}_{\mathrm{it}}+\beta_{0}+\sum_{k=1}^{K}X_{\mathrm{it},k}\beta_{k}+{\mathrm{ICPP}}_{\mathrm{it}}\beta_{k+1}+\mu_{\mathrm{it}}+\nu_{\mathrm{it}}+\varepsilon_{\mathrm{it}}$$

(3) $${\mathrm{GTI}}_{\mathrm{it}}=\sum_{\mathrm{it}}^{\mathrm{NT}}\rho W\times {\mathrm{GTI}}_{\mathrm{it}}+\beta_{0}+\sum_{k=1}^{K}X_{\mathrm{it},k}\beta_{k}+{\mathrm{ICPP}}_{\mathrm{it}}\beta_{k+1}+\mu_{\mathrm{it}}+\nu_{\mathrm{it}}+\varepsilon_{\mathrm{it}}$$

(4) $$\begin{array}{l} {\mathrm{GSDP}}_{\mathrm{it}}=\sum_{\mathrm{it}}^{\mathrm{NT}}\rho W\times {\mathrm{GSDP}}_{\mathrm{it}}+\beta_{0}+\sum_{k=1}^{K}X_{\mathrm{it},k}\beta_{k}+{\mathrm{ICPP}}_{\mathrm{it}}\beta_{k+1}\\ \;\;\;+{\mathrm{GTI}}_{\mathrm{it}}\beta_{k+2}+\mu_{it}+\nu_{it}+\varepsilon_{it} \end{array}$$

### 5.3. Mechanism Analysis Results

This paper used the mediating effect model to test the impact mechanism of the ICPP on GSDP. Among the models, the dependent variables in Model 2 and Model 7 were GSDP, and the dependent variable in Model 6 was GTI. The test results of the mediating effect model are shown in Table 6. In Model 2 and Model 6, we can see that the treatment effect parameter, ICPP, had a significantly positive impact on GSDP and GTI. In Model 7, the coefficient of ICPP and GTI on GSDP both passed the significance test at the 5% and 1% levels, respectively. In addition, the coefficient of ICPP in Model 7 (0.041) was less than that in Model 2 (0.056). Based on the above results, we conclude that GTI had a partial mediating effect on the impact mechanism of the ICPP on GSDP in the Yangtze River Delta urban agglomeration. In other words, the implementation of the ICPP was conducive to promoting urban GTI, improving GSDP.

## 6. Discussion

The main objective of our paper was to explore the role of government innovation support in the process of urban green sustainable development. We viewed the China Innovative City Pilot (ICPP) policy as a quasi-natural experiment and used it as a proxy variable for the intensity of government innovation support in the Yangtze River Delta urban agglomeration. Based on the panel data of 24 cities in the Yangtze River Delta urban agglomeration from 2001 to 2020, we used the SDID model with two-way fixed effects to estimate the impact of China’s ICPP on GSDP. We found that after controlling for time-fixed effects, spatial after the fixed effects, and the influence of control variables, the ICPP had a significant positive impact on green sustainable development performance (GSDP) in the Yangtze River Delta urban agglomeration, which means that the government can promote the level of urban green sustainable development by increasing the intensity of innovation support.

Although some scholars have attempted to assess the policy effects of the ICPP, the frequency of this topic in the international literature remains low. For example, previous scholars have discussed the policy impact of the ICPP on energy productivity [81], financial development [82], and ecological efficiency [83], but few publications have analyzed the policy spillover effects of the ICPP from the perspective of GSDP. The results of the above literature show that the ICPP improved energy productivity, financial development, and ecological efficiency in various regions of China, which means that the implementation of the ICPP can make positive contributions to the sustainable development of the regional economy and environment. Our findings are fully consistent with the main viewpoints of previous scholars and are a useful supplement to the abovementioned findings. However, these studies all used the traditional DID analysis framework, and ignoring the spatial spillover effects may lead to biased identification of the policy effects [84]. Therefore, this article incorporated the spatial lag terms of the explained variables based on traditional DID to identify the policy effects of the ICPP more effectively. According to the empirical results, we found that GSDPs in the Yangtze River Delta urban agglomeration were not only affected by the ICPP but also significantly affected by the GSDP of neighboring cities, which may mean that when neighboring cities adopt strong environmental protection measures, they will also encourage local governments to introduce strong environmental protection policies to improve the overall level of green and sustainable development in the Yangtze River Delta urban agglomeration. Finally, we also found that ICPP can influence GSDP through the channel of GTI, which means that China’s ICPP provides a very favorable innovation milieu for technological innovation. It is well known that the Porter hypothesis argues that appropriate environmental regulation can stimulate enterprises to conduct more innovative activities, thus compensating for the negative impact of environmental protection costs [75]. However, a more appropriate explanation comes from a study by Wang et al. [85], who argued that the location decision of polluting companies will have a significant impact on the GSDP of a city, because the pressure of environmental protection policy will push some polluting enterprises to move to other areas. The spatial redistribution process of a large number of polluting enterprises will promote environmental policies to release greater policy effects through technological innovation. To sum up, the ICPP is a very beneficial institutional arrangement that can realize the coordinated development of the regional economy and ecological environment.

The innovations of this paper and its supplementation of the existing research are as follows. First, this study used the SBM directional distance function based on the undesired outputs to measure green total factor productivity to denote the GSDP of the Yangtze River Delta urban agglomeration. Second, this study combed the literature review of green sustainable development, government innovation support, innovative city, and the interaction among them, in detail, and adopted the SDID model for empirical research to reduce the spatial deviation of the traditional DID model. Third, this study incorporated GTI into the research framework, and through theoretical and empirical analyses studied its mediating effect on the impact mechanism of the ICPP on GSDP in order to break the “black box” between the two. This paper also had some limitations. Firstly, although the evaluation index system established in this study to measure GSDP supplemented the existing research, it is still not very comprehensive. Therefore, in future research, we will continue to improve the measurement index system for GSDP and consider including other resource factor inputs into the evaluation. Secondly, given that the sample in this study was the Yangtze River Delta urban agglomeration, the sample size was not large when compared with all the prefecture-level cities in China. Therefore, we will expand the sample size and study the impact mechanism of the ICPP on GSDP using nationwide urban panel data in future studies.

## 7. Conclusions

Based on the SDID model, this paper evaluated whether and how the ICPP affected the GSDP in China’s Yangtze River Delta urban agglomeration, using whether to become an ICPP pilot city from 2001 to 2020 as a quasi-natural experiment. The research conclusions of this paper are as follows. Firstly, GSDP had a positive spatial spillover effect between neighboring cities—that is, GSDP in the Yangtze River Delta urban agglomeration tended to be clustered at high values with high values or at low values with low values. Secondly, the implementation of the ICPP helped to improve the GSDP in the Yangtze River Delta urban agglomeration, and this conclusion still holds after multiple robustness tests. Thirdly, the results of the mechanism analysis show that the ICPP can improve the GSDP in the Yangtze River Delta urban agglomeration through GTI. Therefore, ICPP is a policy that can effectively enhance the ability of urban sustainable development. Based on the above conclusions, this paper puts forward the following policy implications.

Firstly, the central government should speed up the expansion of the coverage of ICPP pilot cities. Since Shenzhen became the first ICPP pilot city in China in 2008, the construction of an innovative city has been in effect for over 15 years. As of 2022, a total of 103 cities (districts) in China have been approved to be innovative pilot cities, and this policy provides strong support for the sustainable development of these cities. However, the number of ICPP pilot cities currently only accounts for 15.6% of all cities, which is still relatively low. An urban innovation milieu that does not implement the ICPP will be constrained by the old system, which may further widen the gaps in regional technological, economic, and social development. In view of the positive effect of the ICPP on GSDP in the Yangtze River Delta urban agglomeration, it is necessary for more cities to share the policy dividends of the ICPP as soon as possible.

Secondly, local governments should adhere to the role of the market in resource allocation and create a green economic development model with innovation capabilities as the core. The Yangtze River Delta urban agglomeration is the most economically developed and highly concentrated area of innovation resources in China, as well as the most concentrated area of pollution-intensive industrial enterprises. Government departments should give full play to the role of technological innovation in green and sustainable development, promote the innovation and transformation of enterprise sewage technology through market-oriented means, increase R&D investment to stimulate the enthusiasm of enterprises for green innovation, and promote the transformation of green patent achievements into the actual productivity of enterprises. In addition, local governments should also strengthen the assessment of environmental protection in the evaluation of innovative city construction, improve the intensity of environmental regulation, and actively implement fiscal and taxation policies that are conducive to the technological innovation of enterprises.

Thirdly, a strategic alliance for green technology innovation in the Yangtze River Delta urban agglomeration to strengthen the efficiency of collaborative pollution control between regions should be established. The green technology innovation strategic alliance can reduce the transfer cost of new technology and avoid the waste of R&D expenses caused by repetitive technological innovation. At the same time, the green technology innovation strategic alliance is also conducive to the introduction of technology and the sharing of achievements, so that new technologies can be quickly promoted and applied by industrial enterprises on a large scale. In addition, GSDP had a very significant spatial spillover, and it is necessary to strengthen the coordination mechanism of pollution prevention and control in various regions and realize the unified promotion of environmental protection policies among pilot cities by strengthening the exchange and cooperation of management experience between innovative cities. By establishing the linkage mechanism of trans-regional environmental governance, it is possible to break the traditional “territorial management model” and promote the joint prevention and control of waste gas, wastewater, and solid waste between cities to give full play to the spatial spillover effect of innovative city pilot policies on environmental improvement to a greater extent.

## Figures and Tables

**Table 1:** The evaluation index system of GSDP.

Categories	Subsystem	Measurement Index
Input indicators	Labor input	The number of employees in urban units
	Capital input	Fixed asset investment
	Technology input	Science and technology expenditure in government budget expenditure
	Water resources input	Total water supply
	Electric energy input	The whole society’s electricity consumption
Output indicators	Desired output	the regional GDP
		the green area of built-up areas
	Undesired output	Industrial wastewater emissions
		Sulfur dioxide emissions
		Dust emissions

**Table 2:** Results of the descriptive analysis, correlation analysis, and unit root test.

Variables	Symbol	Mean	Standard Deviation	Correlation Coefficient	IPS Test	LLC Test
Green sustainable development performance	GSDP	0.718	0.540	1.000	−8.104 ***	−12.271 ***
The effect of ICPP	ICPP	0.069	0.043	0.075 ***	−1.481 **	−4.634 ***
Foreign direct investment	FDI	0.041	0.022	0.182 ***	−5.316 ***	−8.453 ***
Human capital level	HCL	0.032	0.026	−0.236	−8.537 ***	−10.306 ***
Labor structure	LAS	0.283	0.121	0.053 ***	−2.865 ***	−12.846 ***
Information infrastructure	INI	0.105	0.159	0.109 ***	−1.281 **	−9.629 ***
R&D intensity	RDI	0.073	0.058	0.117 **	−3.359 ***	−5.715 ***
Urbanization level	URL	0.505	0.274	−0.038 **	−6.159 ***	−8.087 ***

Note: **, and *** represent significance at the statistical levels of 10% and 5%, respectively.

**Table 3:** Results of Global Moran’s I of GSDP from 2001 to 2020.

Year	GSDP	Year	GSDP
2001	0.084 ***	2011	0.074 ***
2002	0.059 **	2012	0.057 **
2003	0.066 ***	2013	0.068 ***
2004	0.069 ***	2014	0.087 ***
2005	0.081 ***	2015	0.092 ***
2006	0.073 ***	2016	0.065 ***
2007	0.079 ***	2017	0.070 ***
2008	0.071 ***	2018	0.089 ***
2009	0.085 ***	2019	0.055 **
2010	0.080 ***	2020	0.076 ***

Note: **, and *** represent significance at the statistical levels of 10% and 5%, respectively.

**Table 4:** The benchmark regression results.

	Model 1	Model 2
Variables	GSDP	GSDP
ICPP	0.081 ***	0.056 ***
	(0.026)	(0.019)
FDI		−0.162 **
		(0.080)
HCL		0.088
		(0.132)
LAS		0.127 ***
		(0.034)
INI		0.059 **
		(0.026)
RDI		0.105 ***
		(0.017)
URL		−0.040 *
		(0.023)
_cons	0.184 ***	0.119 **
	(0.042)	(0.056)
spatial fixed effects	YES	YES
time fixed effects	YES	YES
*ρ*	0.182 ***	0.226 ***
	(0.035)	(0.043)
N	480	480
R^2^	0.564	0.703

Note: The figures in parentheses represent the standard error of the respective coefficients. *, **, and *** represent significance at the statistical levels of 10%, 5%, and 1%, respectively. YES: the control variables were added to the regression model.

**Table 5:** The regression results of the robustness checks.

	Model 3	Model 4	Model 5
Variables	GSDP	GSDP	GSDP
ICPP	0.078 ***	0.107 **	0.044
	(0.023)	(0.045)	(0.065)
Constant	0.145 ***	0.203 **	0.171 **
	(0.034)	(0.101)	(0.082)
Control Variables	YES	YES	YES
spatial fixed effects	YES	YES	YES
time fixed effects	YES	YES	YES
Spatial rho	0.138 ***	0.085 ***	0.196 **
	(0.040)	(0.024)	(0.083)
N	480	480	480
Adj-R^2^	0.592	0.655	0.521

Note: The figures in parentheses represent the standard error of the respective coefficients. **, and *** represent significance at the statistical levels of 10% and 5%, respectively. YES: the control variables were added to the regression model.

**Table 6:** The results of the mediating effect model.

Variables	Model 2	Model 6	Model 7
	GSDP	GTI	GSDP
ICPP	0.056 ***	0.131 **	0.041 **
	(0.019)	(0.062)	(0.018)
GTI			0.115 ***
			(0.027)
Control Variables	YES	YES	YES
spatial fixed effects	YES	YES	YES
time fixed effects	YES	YES	YES
*ρ*	0.226 ***	0.281 ***	0.323 ***
	(0.043)	(0.059)	(0.052)
N	480	480	480
R^2^	0.703	0.358	0.741

Note: The figures in parentheses represent the standard error of the respective coefficients. **, and *** represent significance at the statistical levels of 10% and 5%, respectively. YES: the control variables were added to the regression model.

## Data Availability

The data used in this study were sourced from statistical yearbooks; please refer to the second paragraph in Section 3.3 for details.

## Appendix A

**Table A1:** Pilot year and city during 2001–2020 in the Yangtze River Delta urban agglomeration.

Year	Number	City
2010	8	Shanghai, Nanjing, Wuxi, Changzhou, Suzhou, Ningbo, Jiaxing, Hefei
2011	1	Zhenjiang
2012	1	Nantong
2013	5	Yancheng, Yangzhou, Hangzhou, Huzhou, Taizhou
2018	4	Shaoxing, Jinhua, Wuhu, Ma’anshan
